# *Staphylococcus aureus* isolated from selected dairies of Algeria: Prevalence and susceptibility to antibiotics

**DOI:** 10.14202/vetworld.2019.205-210

**Published:** 2019-02-08

**Authors:** Asmaa Manel Matallah, Leila Bouayad, Sofiane Boudjellaba, Faiza Mebkhout, Taha Mossadak Hamdi, Nadjia Ramdani-Bouguessa

**Affiliations:** 1 Laboratory of Food Hygiene and Quality Insurance System (HASAQ), Higher National Veterinary School, Rue Issad Abbes, Oued Smar, Algiers 16000, Algeria; 2 Research Laboratory Management of Local Animal Resources (GRAAL), Higher National Veterinary School, Rue Issad Abbes, Oued Smar, Algiers 16000, Algeria; 3 Laboratory of Medical Biology, Mohamed Mahmoudi City, Number 209 Bis, Baraki, Algiers, Algeria

**Keywords:** antibiotic susceptibility, pasteurized milk, raw milk, *Staphylococcus aureus*

## Abstract

**Aim:**

The objectives of this study were to assess the prevalence of *Staphylococcus aureus* in raw milk in Algerian dairies, to study the effect of seasons on the contamination of milk and the susceptibility of isolated strains to antibiotics, and to estimate the risk on the health consumer.

**Materials and Methods:**

The ISO method 6888-1 (1) was used for *Staphylococcus* screening. Antimicrobial susceptibility to the 11most used antibiotics in veterinary medicine was assessed using the disk diffusion assay.

**Results:**

The overall prevalence was 31.56% (95/301); 34.84% (85/244) from raw milk collectors cisterns (MCC), 22.73% (5/22) from mixing tank milk before pasteurization, and 14.29% (5/35) from pasteurized tank milk (p<0.05). A significant difference (p<0.001) of contamination on MCC was observed between dairies without season influence (p≥0.05). It was observed that 49.47% of *S. aureus* isolates were resistant to penicillin, 5.26% to tetracycline, 4.21% to erythromycin, 3.15% to neomycin, 2.10% to cefoxitin, 2.10% to clindamycin, and 1.05% to ofloxacin. No resistance was observed for vancomycin, gentamicin, chloramphenicol, and trimethoprim-sulfamethoxazole.

**Conclusion:**

A high prevalence of *S. aureus* from MCC was observed without significant effect of season. The pasteurization does not ensure the elimination of bacteria in all samples. Half of the isolates were resistant to penicillin. These findings emphasize the importance of *S. aureus* control in Algerian milk industry at different levels to improve public health.

## Introduction

Zoonoses are infectious diseases that can naturally be transmitted between animals and humans. The severity of these diseases in humans may vary according to the main origin of the infection [1]. Raw milk may contain pathogenic microorganisms; it may occasionally play a role in the transmission of these pathogenic bacteria to humans. Among the most isolated pathogenic bacteria in milk that cause disease, we can cite *Salmonella*, *Brucella, Listeria, Escherichia coli*, and *Staphylococcus aureus* [2]. Some strains of *S. aureus* are the causative agents of foodborne intoxication [3]; their pathogenicity effects are due to the thermostable enterotoxins [4]. Pasteurization eliminates *S. aureus* bacterium, however, once formed, the thermostable enterotoxins make them their biological activity [5,6]. Monitoring the resistance of bacteria of the animal origin allows collecting a set of data to characterize trends, to detect new events at the origin of an alert, to document the level of the resistance of different bacterial species, and, finally, to study the emergence of new serotypes with antibiotic resistance profiles.

The emergence of antibiotic-resistant bacteria has become a global public health concern affecting human and veterinary medicine [7]. In fact, the administration of antibiotics for the feeding of animals for curative purposes or as growth promoters may be a major factor in the selection of antimicrobial-resistant bacteria [8].

The objectives of this study were to assess the prevalence of *S. aureus* in raw milk in dairies, to study the effect of seasons on the contamination of milk and the susceptibility of isolated strains to antibiotics, and to estimate the risk on the health consumer.

## Materials and Methods

### Ethical approval

The raw milk was taken from collectors cisterns, which did not need contact with animals. The present study did not involve any invasive procedure, and hence, ethical approval is not required.

### Sampling

The study was carried out during the period from March 2016 to March 2017 and was realized in three different dairies, two of them located in the Wilaya of Algiers (“A” and “B”) and one in the Wilaya of Boumerdès “C.” In total, 301 samples were collected at three processing steps: (i) At milk reception from different collectors, (ii) after mixing milks before pasteurization, and (iii) after pasteurization. The volumes collected were 5 × 100 ml of milk for each sample according to the Algerian interministerial decree of 2016 fixing the microbiological criteria for foodstuffs N39/2017.

- 244 samples of raw milk were from cisterns of different collectors (milk collectors cisterns [MCC]).
- 22 samples of raw milk were from the mixing tank of different milks before pasteurization (MTMBP).
- 35 samples were from milk tank after pasteurization (TMP).

The samples were collected during the four seasons of this year: 112 samples were collected during the spring, 60 in summer, 57 in fall, and 72 in winter. After aseptic sampling and identification, the samples are quickly refrigerated and transported into a cooler to the food hygiene Laboratory of the Higher National Veterinary School, where they were analyzed the same day.

### Bacteriological analysis

The Baird-Parker agar supplemented with 5% egg yolk and 0.5% potassium tellurite was used for bacteriological isolation according to the ISO 6888-1 method. The putative colonies were tested for catalase, coagulase rabbit plasma and the Pastorex Staph Plus assay (Bio-Rad, Marnes-la-Coquette, France) are considered as *S. aureus* strains, the isolates that responded positively to the three tests.

### Study of susceptibility to antibiotics

The susceptibility of isolated strains was tested with 11 antibiotics belonging to the different classes (β-lactamines, cyclin, aminoglycosides, macrolides, quinolones, glycopeptides, phenicoles, and sulfa) by the disk diffusion method on Mueller-Hinton agar (Conda-Pronadisa, Madrid, Spain), according to the standards [9,10] recommended by the WHO. The antibiotic discs (BioRad, Marnes-la-Coquette, France) used are as follows: Penicillin (10 UI), cefoxitin (30 µg), tetracycline (30 µg), neomycin (30 µg), gentamicin (10 µg), erythromycin (15 µg), clindamycin (2 µg), ofloxacin (5 µg), vancomycin (30 µg), chloramphenicol (30 µg), and trimethoprim + sulfamethoxazole (1.25/23.73 µg).

### Statistical analysis

The results were analyzed statistically using the SPSS software for Windows (version 20.0; SPSS Inc., Chicago, IL, USA). Descriptive statistics and the relationship between contamination samples and different types of samples were analyzed using Chi-square test. The effect of season and milk provenance and milk nature on contamination samples was analyzed using Chi-square analysis. The level of significance was determined at confidence levels of p<0.05.

## Results

From the 301 samples tested, 95 (31.56%) were found positive for *S. aureus*, distributed as follows: 34.83% (85/244) of MCC samples, 22.73% (5/22) of MTMBP, and 14.29% (5/35) of TMP (p<0.05), respectively. The MCC samples collected from the three dairies A, B, and C had contamination rates of 14.29% (3/21), 26.47% (36/136), and 52.87% (46/87), respectively (Table-1). The MTMBP showed the levels of contamination for the three dairies A, B, and C of 10% (1/10), 28.57% (2/7), and 40% (2/5), respectively. The TMP collected from the dairies A and B showed contamination rates of 18.18% (2/11) and 23.08% (3/13), respectively. No TMP contamination was recorded in dairy C.

**Table-1:** Contamination of milk (MCC, MTMBP, and TMP) by *S. aureus* according to dairies and season.

Parameters	Total (n)	Positive (number of people)	Positive (%)	p-value
MCC				
Dairy A	21	3	14.29	<0.001
Dairy B	136	36	26.47	
Dairy C	87	46	52.87	
MTMBP				
Dairy A	10	1	10	NS
Dairy B	7	2	28.57	
Dairy C	5	2	40	
TMP				
Dairy A	11	2	18.18	NS
Dairy B	13	3	23.08	
Dairy C	11	0	0	
MCC				
Fall	41	14	34.15	NS
Summer	50	20	40.00	
Winter	58	14	24.14	
Spring	95	37	38.95	
MTMBP				
Fall	7	3	42.86	NS
Summer	3	1	33.33	
Winter	6	0	0.00	
Spring	6	1	16.67	
TMP				
Fall	9	0	0.00	NS
Summer	7	2	28.57	
Winter	8	0	0.00	
Spring	11	3	27.27	
Total	301	95	31.56	

**NS=Not significant (p≥0.05), *S. aureus*=*Staphylococcus aureus*, MCC=Milk collectors cisterns, MTMBP=Mixing tank of different milks before pasteurization

The rates of the contamination of MCC samples recorded during the spring, summer, fall, and winter season were 38.94% (37/95), 40% (20/50), 34.15% (14/41), and 24.13% (14/58), respectively (p≥0.05). MTMBP contamination rates recorded during three seasons, spring, summer, and fall were 16.67% (1/6), 33.33% (1/3), and 42.86% (3/7), respectively. TMP samples showed contamination rates during the two seasons, spring and summer of 27.27% (3/11) and 28.57% (2/7), respectively. No contamination was recorded during the fall and winter. The MCC samples from dairy A showed no contamination during three seasons, spring, fall, and winter, but the rate of contamination recorded during the summer season was 75% (3/4). In the same dairy, no contamination was also recorded in MTMBP and TMP during the same three seasons, but the rates of contamination recorded during the summer were 50% (1/2) and 66.67% (2/3), respectively. The MCC from the dairy B showed the levels of the contamination of 36.21% (21/58), 20.83% (5/24), 20% (4/20), and 17.65% (6/34) during the spring, summer, fall, and winter seasons, respectively. A contamination rate of the MTMBP samples of 66.67% (2/3) was recorded only during the fall. TMP samples recorded a contamination rate of 60% (3/5) during spring, and no contamination was recorded in other seasons. In dairy C, the levels of the contamination of MCC samples were 50% (16/32), 54.55% (12/22), 62.50% (10/16), and 47.06% (8/17) during spring, summer, fall, and winter, respectively. The levels of MTMBP contamination were 50% (1/2) and 100% (1/1) for spring and fall seasons, respectively. TMP did not present any contamination during the four seasons.

### Susceptibility of strains to antibiotics

A total of 41.05% of tested strains were sensitive to all tested antibiotics; 47.36% (45/95) had monoresistance, including 3.15% (03/95) to erythromycin, 1.05% (01/95) to ofloxacin or tetracycline, and 42.10% (40/95) to penicillin. Double resistance was observed in 4.21% (04/95) of the isolates, two to neomycin and penicillin, one to cefoxitin and penicillin, and one to tetracycline and penicillin. Multiple antibiotic resistance was observed in 3.15% (03/95) of the isolates, among which one included resistance to cefoxitin, neomycin, and penicillin. The remaining two of isolates coming from the same collector sampled in two different seasons (summer and winter) were resistant to tetracycline, erythromycin, clindamycin, and penicillin. Intermediate susceptibility was observed in 7.36% (07/95) of the isolates: Trimethoprim-sulfamethoxazole, clindamycin, neomycin, or ofloxacin. One of them showed intermediate susceptibility to two antibiotics (trimethoprim-sulfamethoxazole and ofloxacin) (Table-2). The overall resistance rates were 49.47% for penicillin, 5.26% for tetracycline, 4.21% for erythromycin, 3.15% for neomycin, 2.10% for cefoxitin, 2.10% for clindamycin, and 1.05% for ofloxacin. No resistance was observed for vancomycin, gentamicin, and chloramphenicol. The intermediate sensitivity was observed for the following antibiotics: 1.05% for clindamycin, 5.26% for trimethoprim + sulfamethoxazole, 1.05% for ofloxacin, and 1.05% for neomycin (Table-3).

**Table-2:** Resistance profile of *S. aureus* isolates to antibiotics.

Number of stains	Sensitive to all ATB (%)	Single resistance (%)	Double resistance (%)	Multiple (≥3) résistance (%)	Intermediate sensitivity (%)
95	39 (41.05)	45 (47.36)	4 (4.21)	3 (3.15)	7 (7.36)

*S. aureus*=*Staphylococcus aureus*

**Table-3:** Susceptibility of *S. aureus* isolates to tested antibiotics.

Antibiotics	Break points (mm)	Sensible (%)	Intermediate (%)	Resistant (%)
TE	14-19*	90 (94.73)	0	5 (5.26)
E	13-23*	91 (95.78)	0	4 (4.21)
CM	14-21*	92 (96.84)	1 (1.05)	2 (2.10)
SXT	10-16*	90 (94.73)	5 (5.26)	0
OFX	14-18*	93 (97.89)	1 (1.05)	1 (1.05)
VA	≥15**	95 (100)	0	0
GM	12-15*	95 (100)	0	0
N	13-18*	91 (95.78)	1 (1.05)	3 (3.15)
C	12-18*	95 (100)	0	0
P	28-29*	48 (50.52)	0	47 (49.47)
FOX	21-22*	93 (97.89)	0	2 (2.10)

C=Chloramphenicol, CM=Clindamycin, E=Erythromycin, FOX=Cefoxitin, GM=Gentamicin, N=Neomycin, OFX=Ofloxacin, *P*=Penicillin, SXT=Trimethoprime+Sulfamethoxazole, TE=Tetracycline, VA=Vancomycin,

* *CLSI* [9],

** *CLSI* [10],

*S. aureus*=*Staphylococcus aureus*

## Discussion

*S. aureus* is considered to be the most common mammary pathogen found in bovine mastitis in the whole world and is an obvious contributor to milk contamination [11]. In the present study, the MCC was the most contaminated milk type presenting rate contamination of 34.84%. This rate was higher than that recorded in previous studies in Western of Algeria [12] and the central region of our country [13,14] with rates of 26.25%, 18%, and 29.81%, respectively. It is higher than that of recorded in Iran (21%) [15] and is also significantly higher than rates noted in India (6.00%) [16] and in Brazil (7.03%) [17]. This difference can be attributed to the fact that the most of these researchers carried out their samples on farms, while our study was interested in the milk of collectors who underwent several means of transport and manipulations before arriving at the dairy from where they were taken. The same rate is lower than that noted by Hamiroune *et al*. [18] (58.5%) who evaluated the bacteriological quality of raw milk at various stages of the production chain on farms in Algeria and that of in Brazil (68%) [19]. It is also lower than the rate found in Northern Morocco (40%) [20]. Milk is normally sterile if the cow does not suffer from clinical or subclinical mastitis [21]. Contamination may come from the udder itself or the non-observance of hygiene rules during milking, such as a deficiency of hygiene in hands, teat, and storage tanks [22]. Insufficient cleaning of milking machines and the use of contaminated water during milking would result in the persistence of *S. aureus* in raw milk [23]. This milk represents a danger in the dairy industry, as the production of staph toxin resistant to the thermal pasteurization process is normally found at a temperature of 40-45°C [24], although Smith [25] detected the production of toxins at a temperature of 10-46°C. It was observed in this study that the milk refused at the level of the dairies were delivered to the points of sale to be resold on their own or converted into curdled milk and butter made in artisanal way, knowing that many people prefer consumption of cow’s milk in the raw state [23]. The counts of *S. aureus* above 10³ increase the likelihood of the production of boiling resistant staphylococcal toxins produced in households during the purchase of raw milk and the pasteurization process [26]. In our study, pasteurized milk showed a contamination rate of 14.29% (5/35). These results are lower than those observed by Freitas *et al*. [27] and De Oliveira *et al*. [19] with rates of 30.5% and 30%, respectively. The persistence of *S. aureus* in pasteurized milk is probably related to the high initial charge of raw milk, the mixing of milk from several sources would promote cohabitation and the appearance of bacterial strains that are increasingly resistant to heat treatments. Pasteurized milk can also be predisposed to the probable production of the toxin cited by Chapaval *et al*. [28] because, in various situations, store owners turn off the chillers at night to save electricity, leaving the product exposed to the temperature variations [19]. In our study, the observed differences between dairies for the MCC would be due to poor hygiene conditions in dairy farms [29], or most probably due to the presence of subclinical mastitis that often passes unperceived, it may be due also to differences in practices in each dairy. This contamination would depend on the processing capacity of each dairy which could have a negative impact on compliance with the hygiene rules, this even in the presence of an informed control system. This situation results in a very long stay of milk in tanks before heat treatment. We also noted a higher frequency of collection for the dairy “C” (spread out over all the day) with several round trips, while in the other dairies, milk collection was held only early in the morning.

The results of our study also show that the contamination of MCC by *S. aureus* is not significantly different in terms of the seasons. However, it remains important and relatively equivalent during the three-quarters of the year (spring, summer, and fall) with a contamination rate of 38.94%, 40%, and 34.16%, respectively. Indeed, the North of Algeria is known for its high temperatures during this period favorable to the development of *S. aureus*. Effectively, episodes of temperature rise and close cohabitation of the animals in winter would explain the contamination noted even in winter (24.13%). Chapaval *et al*. [28] showed that the production of staphylococcal enterotoxins in milk is noted when it is stored at a temperature of 37°C-42°C or when exposed to temperature fluctuations [28]. The study on UHT cow’s milk deliberately contaminated with *S. aureus* showed significant development of *S. aureus* in milk incubated at 22°C and 32°C compared to that incubated at 12°C and 18°C [30].

During the summer period, dairy “A” was the most contaminated (75%). This could be explained by the proliferation of *S. aureus* due to heat and their ability to form biofilm in collecting and storage tanks and their resistance to the insufficient cleaning. Knowing that there is only one collector that supplies this dairy and the milk collected in this dairy is used for the manufacturing of fermented milk, which can be contaminated because the growth of *S. aureus* at a low pH depends on the level of contamination initial, modalities of preparation, and the temperature of curdling [30].

The present study showed that 47.36% of isolates had resistance to a single antibiotic, 4.21% to two antibiotics, and 3.15% to several antibiotics. The result of this study was different from those obtained by Chaalal *et al*. [31] for single and multiple resistance (5.3% and 83.9%). They are almost similar for double resistance (6%). Two strains isolated in the same collector, isolated in two different seasons (summer and winter) had multiple resistance for the same antibiotics (tetracycline, erythromycin, clindamycin, and penicillin), this could be explained either by the persistence overtime of these resistant strains in a poorly washed material or in the udder after poorly treated mastitis. Our study showed that 49.47% of the strains studied were resistant to penicillin and 5.26% to tetracycline. Our results are lower than those observed in Algeria [12] (80.95% for penicillin and 71.43% for tetracycline). This difference could be because of this last analyzed milk from subclinical mastitis. They are also lower than those noted by Thaker *et al*. [16] (100%) for penicillin. Our results are higher than those obtained in Malaysia [32] for penicillin 23.3% but similar for tetracycline 5.0% and chloramphenicol and gentamicin 00%. They also reported the lowest rates of 15% to cefoxitin, 10% to clindamycin, and 8.3% to erythromycin. Liu *et al*. [33] observed resistances higher than ours for clindamycin 35.2%, sulfamethoxazole-trimethoprim 20.4%, erythromycin 46.3%, and chloramphenicol 7.41%. The high percentage of resistant to penicillin *S. aureus* isolates may be due to previous contact with these antibiotics, particularly in the treatment of mastitis in dairy farms [34]. According to some authors, resistance is exacerbated by the abusive and systematic use of intramammary injections, especially during the drying period [35]. In Algeria, beta-lactams including penicillin G are largely used in the treatment of mastitis without any susceptibility testing of the bacterial strain against these antimicrobials [12]. The presence of antibiotic resistance in raw milk has the effect of weakening the efficacy of the antibiotic in the treatment of *S. aureus* infections in the consumer. The dissemination of resistant bacteria between different hosts can occur by contamination of food, air, or water. When it reaches a new host, the bacterium can colonize or infect it. It can then disseminate its genes of resistance to other bacteria and also receive genes of resistance from other bacteria [36].

## Conclusion

In this study, the MCC has a high rate of positive samples for *S. aureus*. This contamination provides information on the health status of dairy cows and the lack of hygiene in livestock farms.

The persistence of *S. aureus* in pasteurized milk should attract attention to palliate this problem which could quickly become a plague for public health. High temperatures in our country and episodes of temperature rise, as well as permanent stalling during the winter period, seem to have no significant influence on the development of *S. aureus*. More than half of the strains isolated from *S. aureus* had intermediate susceptibility or resistance to one or more antibiotics, this result would result from the abusive and inappropriate use of these antibiotics in dairy farms and could be a serious problem for human health.

## Authors’ Contributions

AMM designed and conducted the work. AMM and FM were responsible for sample collection and antibiogram analysis. AMM and LB contributed equally to isolate and identify *S. aureus*. LB and SB carried out the statistical analysis. The manuscript was drafted by AMM under the guidance of TMH and NRB. All authors read and approved the final manuscript.
